# Preoperative Chemoradiation in Locally Advanced Rectal Cancer: Efficacy and Safety

**DOI:** 10.14740/gr681w

**Published:** 2015-12-31

**Authors:** Evangelia Peponi, Vlassios Skloupiotis, Dimitris Tsironis, Ifigenia Tasiou, Antonio Capizzello, Chris Tsironis, Konstantinos E. Tsimoyiannis, Evita Pitouli, Evangelos Tsimoyiannis, Pericles Tsekeris

**Affiliations:** a Department of Radiation Oncology, University Hospital of Ioannina, Ioannina, Greece; b Department of Surgery, “Hatzikosta” Community Hospital, Ioannina, Greece

**Keywords:** Rectal cancer, Preoperative chemoradiation, Sphincter preservation

## Abstract

**Background:**

Preoperative chemoradiation (CRT) is considered the standard of care in the management of stage II/III rectal cancer. The aim of this retrospective study was to assess the efficacy and safety of preoperative CRT in our patient cohort with locally advanced rectal adenocarcinoma.

**Methods:**

Forty patients with cT3-4N0-2M0 adenocarcinoma of the lower (n = 26) and mid/upper (n = 14) rectum were enrolled in this study between 2001 and 2012. Radiotherapy (RT) was given to the pelvis. The median prescribed dose was 45 Gy (daily dose, 1.8 - 2.0 Gy). All patients received chemotherapy concurrently with RT and underwent surgery 6 - 8 weeks after CRT. Low anterior resection (LAR) was achieved in 21 patients. Total mesorectal excision (TME) was performed in 24 patients.

**Results:**

Tumor downstaging (expressed as TN downstaging) was observed in 15 patients (38%); a pathological complete response (pCR) was pathologically confirmed in six of them. In nine out of the 26 (23%) patients with low lying tumors, sphincter preservation (SP) was possible. SP was also possible in all but one patient (13%) who achieved a pCR. In three out of 15 patients (8%) with preoperative sphincter infiltration, SP was achieved. With a median follow-up of 58 months, the 4-year local control (LC), distant metastases-free survival (DMFS), disease-free survival (DFS) and overall survival (OS) rates were 89.7%, 86.9%, 79.5% and 81.2%, respectively. The pretreatment tumor size was predictive of response to preoperative CRT. The response to preoperative CRT did show a significant impact on DFS and on OS. TME resulted in a statistically significant increased DFS rate. No grade 3/4 acute toxicity was reported. Three patients developed grade 3 late side effects.

**Conclusion:**

Preoperative CRT demonstrates encouraging rates of disease control and facilitates complete resection and SP in advanced rectal cancer with acceptable late toxicity.

## Introduction

Rectal adenocarcinoma is a common malignancy, especially in developed countries. Together with malignant tumors affecting the colon, colorectal cancer ranks as the third most common cancer in the world.

The combination of postoperative radiotherapy (RT) and 5-fluorouracil-based chemotherapy for locally advanced rectal cancer has been shown to reduce local recurrence and to improve survival compared with surgery alone or surgery plus RT. Over the past decade, there has been a shift in the sequencing of chemoradiation (CRT) relative to surgical resection in clinically staged locally advanced rectal carcinoma after the German randomized study, which demonstrated the superiority of preoperative CRT over postoperative CRT, with improved local control (LC), improved functional results, and lower rates of acute toxicity [1]. The correlation of the level of downstaging after CRT given preoperatively with its ability to achieve better disease control is yet to be described.

The aim of this retrospective study was to present our experience and assess the efficacy and safety of preoperative CRT in patients with locally advanced rectal adenocarcinoma. We have also tried to correlate prognostic factors regarding endpoints like tumor shrinkage, sphincter preservation (SP) and disease control.

## Materials and Methods

### Patient and disease characteristics

Preoperative CRT was administered to 40 patients with histologically confirmed locally advanced rectal adenocarcinoma treated from April 2001 to May 2012 at the University Hospital of Ioannina. Patients excluded from the analysis were those who did not undergo resection (n = 9), received inadequate doses of radiation (< 45 Gy) (n = 5) or had transrectal tumor excision (n = 2). Locally advanced rectal cancer was defined as tumor extension through the bowel wall (i.e., cT3, cT4), and/or node positive tumors, without distant metastases.

All patients underwent pretreatment workup with a complete history, physical examination, proctoscopy and colonoscopy, abdominal/pelvic computerized tomography (CT) and/or pelvic MRI and chest X-ray. All tumors were located at least 1 cm above the anorectal ring, as measured by digital exam. Laboratory tests were performed to evaluate hematologic, renal, and hepatic function.

### Preoperative staging

The preoperative TN staging was determined clinically from physical examination, taking into account the distance from anal verge, and pelvic CT or magnetic resonance imaging (MRI). Transrectal endoscopic ultrasound (EUS) was additionally performed in 21 patients to form and optimize the surgical decisions. In case of discrepancy, the highest T stage was used. TN staging is outlined in Table 1.

**Table 1:** TN Stage Distribution in All Patients

	cN0	cN1	cN2	Total
cT3	23	3	1	27
cT4	10	1	2	13
Total	33	4	3	40

The median tumor size prior to the start of RT was 4 cm (range: 1 - 12 cm). The median distance from the anal verge was 5 cm (range: 1 - 15 cm). Table 2 shows tumor characteristics, regarding distance from anal verge and sphincter infiltration.

**Table 2:** Tumor Characteristics

Tumor characteristics	Number of patients	%
Site of primary tumor		
Upper rectum (> 10 cm from anal verge)	5	13
Middle rectum (5 - 10 cm from anal verge)	9	23
Lower rectum (≤ 5 cm from anal verge)	26	65
Sphincter infiltration	15	38

### Treatment characteristics

#### RT

The radiation was delivered with 6 MV photons using a four-field technique (box technique). Patients were treated in the prone position. A median total dose of 45 Gy (range, 45 - 50.4 Gy) was administered to the planning target volume (PTV). The prescribed dose encompassed at least 95% of the PTV. In four patients, a boost dose of 5.4 Gy to the tumor was added, due to bulk of disease. Portal films were obtained once a week or more often, if clinically indicated. The clinical target volume (CTV) encompassed the primary tumor, adjacent mesorectal tissue, and internal iliac and presacral lymph nodes up to the L5-S1 level. If there was tumor infiltration in other organs of the pelvis anteriorly, the CTV was adjusted to include the involved area with the external iliac lymph nodes.

#### Chemotherapy

For patients receiving capecitabine and RT (n = 27), the capecitabine was administered continuously throughout the course of RT. The dose was 825 mg/m^2^ given twice daily, 7 days per week. For those patients who received 5-FU (n = 13), it was administered as a 6-h daily infusion (400 mg/m^2^ per day) with leucovorin modulation (20 mg/m^2^ per day) for 4 days on week 1 and 5 of RT.

#### Surgery

All patients underwent surgery 6 - 8 weeks after CRT. Prior to the start of the treatment, the option of SP surgery was evaluated taking into account the distance from the lower pole of the primary tumor to the anal verge measured at baseline and after CRT.

The extent of residual tumor in the surgical specimen was staged according to the American Joint Committee on Cancer TNM staging system. A pathological complete response (pCR) was defined as the absence of any viable residual tumor cell in the resected primary tumor and adjacent lymph nodes (Mandard TRG System).

#### Toxicity assessment

Normal tissue effects were graded according to the Radiation Therapy Oncology Group (RTOG)/European Organization for Research and Treatment of Cancer (EORTC) radiation morbidity scoring criteria [2].

### Follow-up

All patients were clinically assessed at regular weekly intervals during the course of irradiation, and 2 - 3 weeks following the completion of RT. After surgery, patients were seen in routine follow-up at least every 3 - 4 months for the first 2 years, every 6 months for the next 3 years, then yearly. At each visit, physical examination, liver function tests and carcinoembryonic antigen (CEA) were obtained. A chest X-ray, an abdominal ultrasound and colonoscopy were performed yearly. Abdominal/pelvic CT was obtained when indicated.

### Statistical analysis

Statistical calculations of Kaplan-Meier curves were performed using StatView^®^ program (Abacus Concepts Inc., Berkeley, CA). A P value of ≤ 0.05 was considered statistically significant.

## Results

### Patients/disease downstaging

Of the 40 analyzed patients, 25 were men and 15 were women. Mean age at surgery was 64 years (range, 32 - 86 years). All patients had a Karnofsky performance status of 80-100%. Median follow-up of the cohort was 58 months (range, 3 - 134 months).

The mean number of lymph nodes (LNs) excised was 12 (range, 3 - 22). The mean number of positive LNs in the surgical specimen was 1.3 (range, 0 - 16).

Clinical response was assessed preoperatively by digital examination. Tumor downstaging was defined by a comparison of the pretreatment TN stage (determined by clinical and radiographic evaluation) to the pathologic stage. Tumor downstaging was observed in 15 patients (38%); a pCR was pathologically confirmed in six of them. CR was defined as absence of viable adenocarcinoma cells in the surgical specimen (ypT0N0). All patients with pCR were alive without evidence of disease at the time of the analysis (median follow-up 61 months (range, 18 - 84)). Table 3 outlines the rates of response to preoperative CRT.

**Table 3:** Rates of Response to Neoadjuvant Chemoradiation

Response	Number of patients	%
pCR	6	15
pPR	7	23
pSD	19	48
Upstaged	6	15

pCR: pathological complete response; pPR: pathological partial response; pSD: pathological stable disease.

The pretreatment tumor size (< 3 cm vs. ≥ 3 cm) was predictive of response to preoperative CRT (expressed as TN downstaging) (P = 0.027). Nodal status, either clinical or pathologic, failed to predict for tumor response.

### SP

For the entire cohort, SP was possible in 21 patients (53%); 19 patients (48%) underwent abdominoperineal resection (APR). Total mesorectal excision (TME) with complete mesorectal fascia excision was performed in 24 patients (60%). In nine out of the 26 (23%) patients with low lying tumors, SP was possible. SP was also possible in all but one patient (13%) who achieved a pCR. In three out of 15 patients (8%) with preoperative sphincter infiltration, SP was achieved.

### Treatment response and disease outcomes

**Figure 1:**
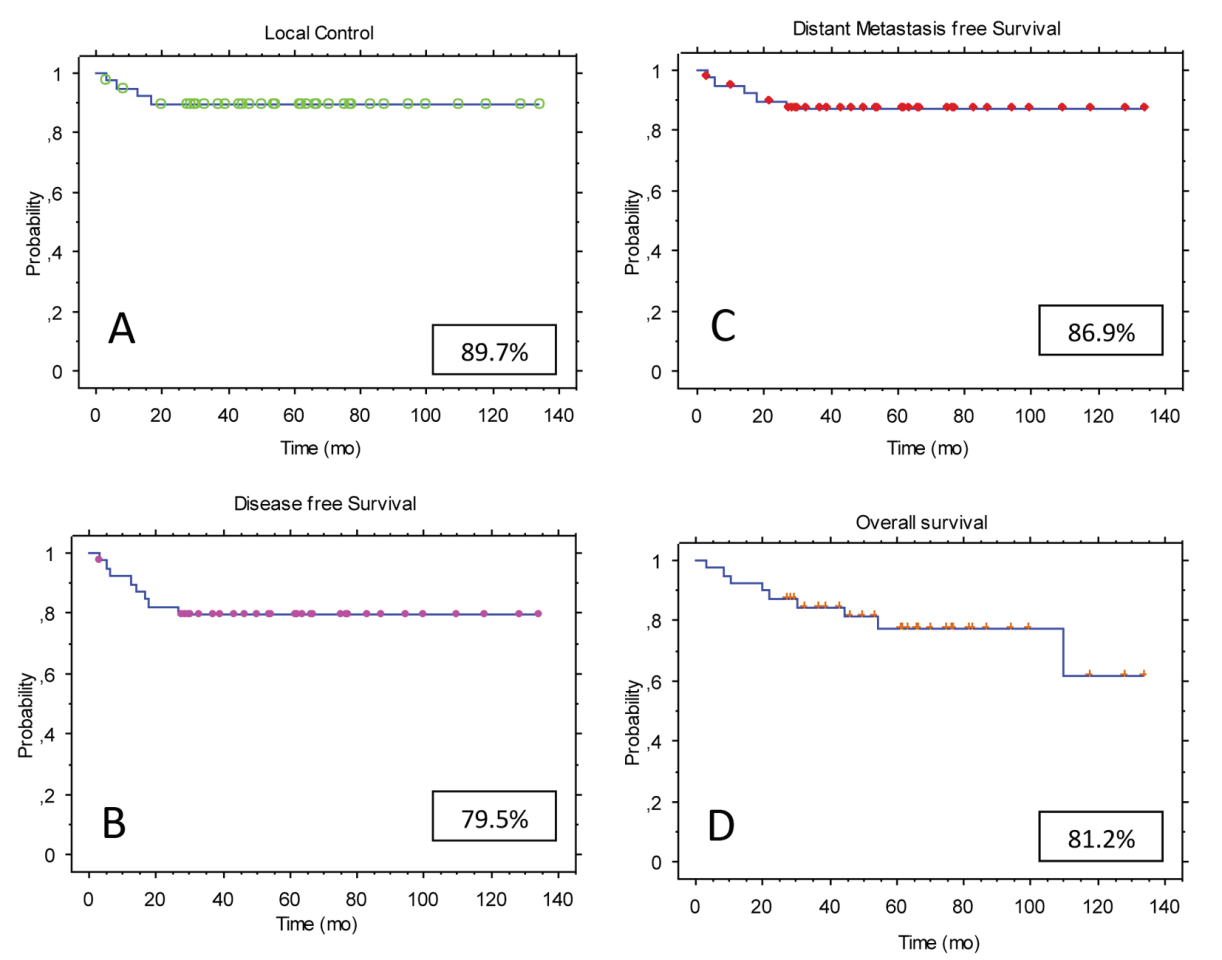
Kaplan-Meier estimates for the study population (n = 40): (A) local control (LC); (B) disease-free survival (DFS); (C) distant metastases-free survival (DMFS); (D) overall survival (OS).

At the time of this analysis, among the 40 patients, 28 were alive without evidence of disease and two were alive with distant metastatic disease. One patient was alive with local recurrence (involvement of the vagina). Nine patients have died, five of them as a result of their disease. With a median follow-up of 58 months (range, 3 - 134), the estimated 4-year LC rate for the entire study population was 89.7% (Fig. 1A). The 4-year disease-free survival (DFS) rate was 79.5% (Fig. 1B), while the distant metastases-free survival (DMFS) rate was 86.9% (Fig. 1C). The actuarial 4-year overall survival (OS) rate was 81.2% (Fig. 1D).

### Toxicity

In the majority of patients, mild diarrhea and tenesmus were observed mostly during second and third week of treatment. Symptomatic medication was given. No treatment interruption due to acute side effects was observed.

Grade 3/4 late radiation-induced complications were seen in three out of 40 patients (8%). Rectal bleeding was reported in two patients (11 months post-RT), while one patient developed a rectovesical fistula (12 months post-RT). In one patient, an APR was conducted 1 year after the LAR, because of stenosis in the anastomosis, which was thought to be a relapse, but the pathology showed a benign stenosis.

### Potential prognostic factors

Univariate and multivariate analyses were performed to examine the impact of various prognostic factors on LC, DFS and OS.

In the univariate analysis, patient-specific factors studied, namely age and gender, were not predictive of LC, DFS and OS. LN involvement, either clinical or pathological, as well as the number of LNs excised had no impact on LC, DFS and OS. Median OS for pathologic node negative cases was 61.8 months as compared to 48.4 months for pathologic node positive patients, hence without reaching statistical significance (P = 0.09).

**Figure 2:**
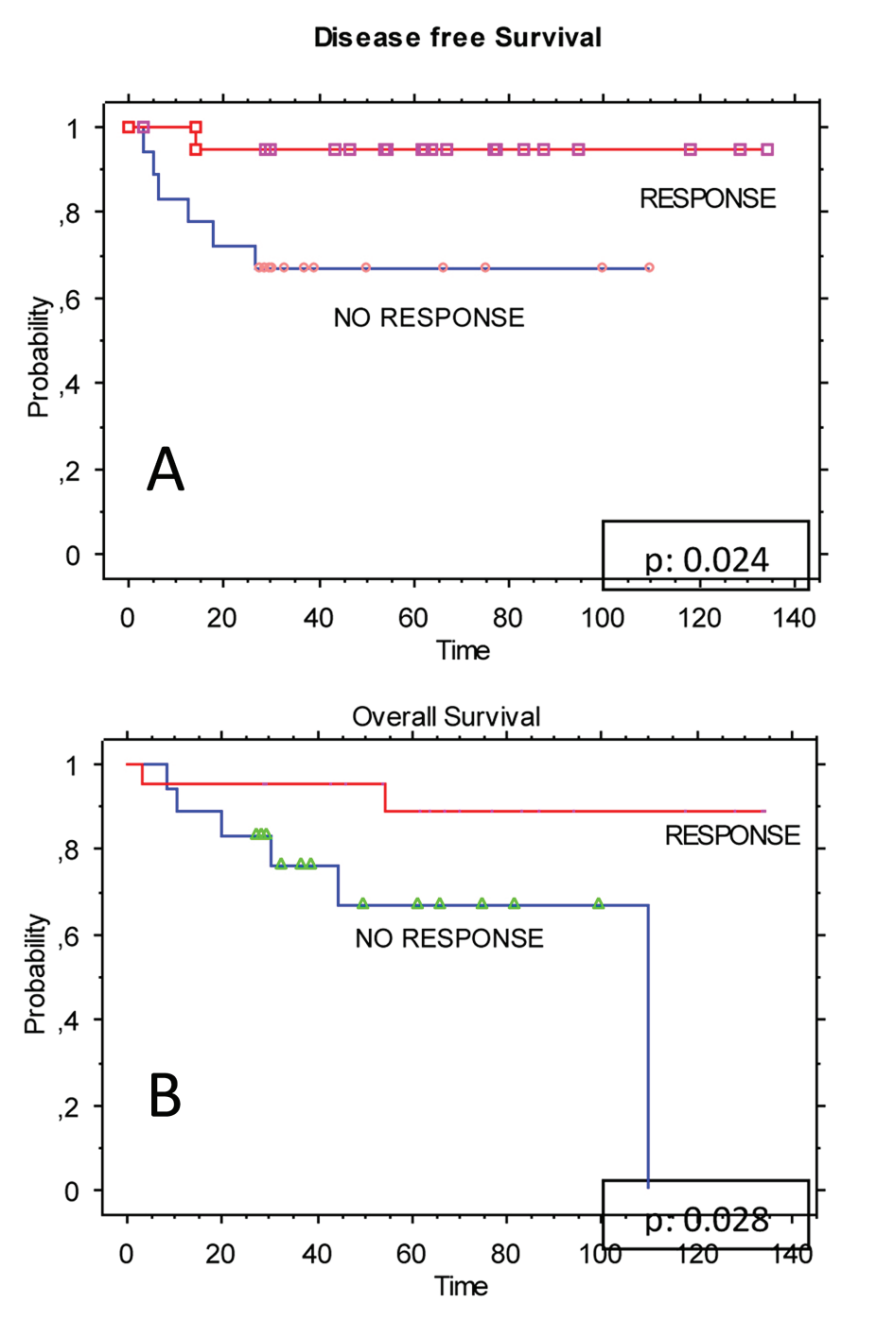
Kaplan-Meier estimates for the study population (n = 40); (A) disease-free survival (DFS) stratified by the response to neoadjuvant CRT; (B) overall survival (OS) stratified by the response to neoadjuvant CRT.

**Figure 3:**
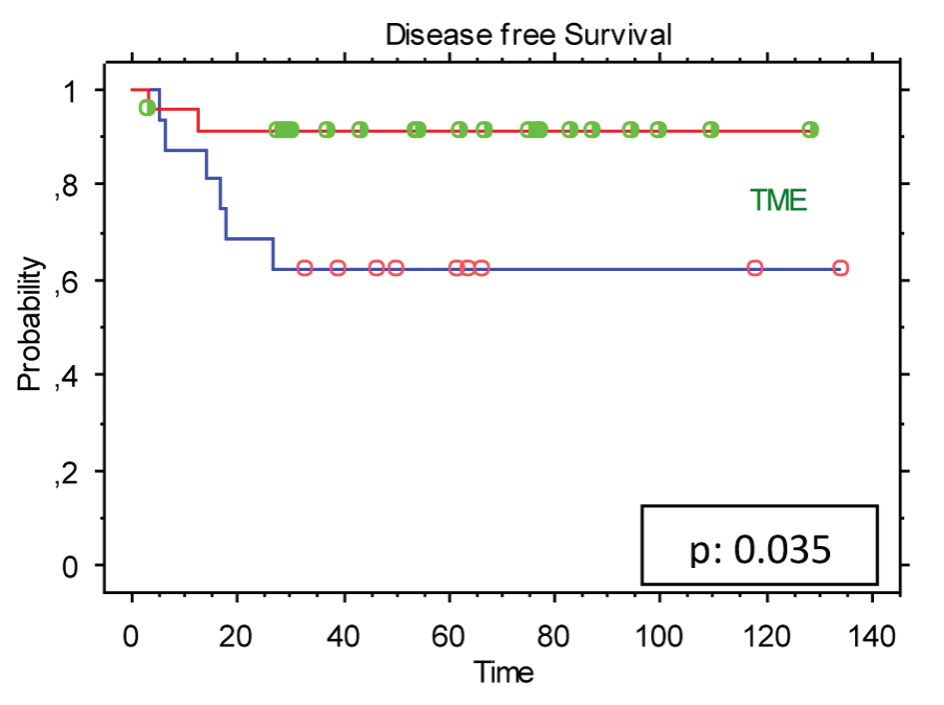
Kaplan-Meier estimates for the study population (n = 40): disease-free survival (DFS) stratified by the total mesorectal excision (TME).

Extent of response to preoperative CRT (expressed as TN downstaging) did show a significant impact on DFS (P = 0.024) (Fig. 2A) and on OS (P = 0.028) (Fig. 2B). Moreover, TME resulted in a statistically significant increased DFS rate (P = 0.035) (Fig. 3).

In the multivariate analysis, response to neoadjuvant CRT significantly affected DFS (P = 0.044) as well as TME showed a favorable impact on DFS (P = 0.052).

## Discussion

The current study, designed to address long-term follow-up (median of 58 months), represents data analysis of experience with preoperative CRT in patients with locally advanced rectal adenocarcinoma, in a clinically homogeneous population uniformly treated at a single institution.

Preoperative CRT results in a better LC rate and a decreased rate of acute and late adverse events, as compared with postoperative CRT [1]. It has been reported that the addition of chemotherapy to preoperative radiation results in a further improvement in the LC rate. However, despite increased statistical power, no significant improvement in DFS or OS was seen [3]. According to our results, patients with pathological pCR did not correlate with improved DFS and OS. However, in accordance to other reported data [4, 5], an excellent prognosis was observed, as the patients with pCR had 100% 5-year actuarial DFS at a median follow-up of 61 months.

It has been suggested that the low abdominal resection (LAR) rates increase when preoperative irradiation is combined with chemotherapy, as compared to preoperative irradiation alone [6]. Thus, preoperative CRT would not only improve LC, but also enhance SP surgery [7]. However, two phase III randomized studies have failed to show a statistically significant impact regarding the rate of SP surgery after neoadjuvant CRT compared to RT alone [8, 9]. Nevertheless, in a recent pooled analysis of these randomized studies, it is suggested that preoperative CRT improves in a modest way the pCR rate parameters (+8%) and the feasibility of R0-R1 resections (+2%) [3]. Trials on preoperative treatment of operable rectal cancer were historically prompted to investigate the possibility of allowing a sphincter-saving procedure in low lying tumors. In our study, performed in a non-selected population, the 53% rate of sphincter-sparing surgery could be considered satisfactory - though inferior - compared to other reported studies [1, 5]. In fact, SP was possible in nine out of the 26 (23%) patients with low lying tumors (≤ 5 cm from anal verge) and in three out of 15 patients (8%) with preoperative sphincter infiltration at presentation.

With a median follow-up of 58 months (range, 3 - 134), the estimated 4-year disease control of our analysis was comparable to other reported studies [9, 10].

Achievement of a complete mesorectal excision with all margins (including circumferential) disease-free is the cornerstone of the treatment of rectal cancer [11] and this objective is often given by neoadjuvant CRT [12, 13]. Our results, in accordance to other studies, confirm that TME technique is superior to the standard surgical procedure, at least regarding DFS.

Pretreatment stage and tumor characteristics did not affect the clinical end points. Only tumor size had a statistically significant positive impact on response to preoperative CRT (expressed as TN downstaging) (P = 0.027), with a cutoff of 3 cm, while in a long-term analysis of 165 patients by Valentini et al [14], a cutoff of 6 cm was suggested.

The basic limitation of the present study is its retrospective design. It is therefore limited by the bias inherent in this type of analysis. Nevertheless, patients were treated relatively consistently and data were collected with meticulous follow-up. Additionally, although the imaging studies followed standardized protocols, it may be that the expertise varied among the individuals performing and interpreting these studies, resulting perhaps in a preoperative staging variation.

### Conclusion

In conclusion, based on the durability of our study results beyond a median follow-up of 4 years, patients with advanced rectal cancer who undergo preoperative CRT followed by complete surgical resection of either a node-negative or node-positive specimen can anticipate excellent long-term DFS and OS with acceptable toxicity. The quality of staging (MRI), planning and delivery of radiation, surgery (TME) and pathological reporting in clinical practice may refine the current preoperative CRT strategies, and will hopefully significantly impact response rates and survival.
